# Adoption of Deep Learning Methods for SSVEP Classification in XR-Based Wearable Brain–Computer Interfaces

**DOI:** 10.3390/s26165102

**Published:** 2026-08-12

**Authors:** Leopoldo Angrisani, Egidio De Benedetto, Andrea De Maria, Luigi Duraccio, Annarita Tedesco

**Affiliations:** 1 Department of Electrical Engineering and Information Technology (DIETI), University of Naples Federico II, Via Claudio n. 21, 80125 Naples, Italy; angrisan@unina.it (L.A.); andrea.demaria@unina.it (A.D.M.); luigi.duraccio@unina.it (L.D.); 2 Department of Public Health, University of Naples Federico II, Via Pansini, 80131 Naples, Italy; annarita.tedesco@unina.it

**Keywords:** augmented reality, brain–computer interface (BCI), deep learning, extended reality (XR), measurement, steady-state visually evoked potential (SSVEP), uncertainty, wearable systems

## Abstract

This paper addresses steady-state visually evoked potential (SSVEP) classification in wearable extended-reality (XR) brain–computer interfaces (BCIs), with a threefold objective. First, it investigates the effectiveness of deep learning (DL)-based SSVEP classification under XR stimulation, where platform-dependent rendering, optical see-through visualization, reduced luminance contrast, and interaction with the real environment may degrade the quality of the elicited EEG response. Second, a *metrology-based* performance assessment is proposed according to the Guide to the Expression of Uncertainty in Measurement (GUM), with classification accuracy and information transfer rate (ITR) expressed as best estimates with associated standard uncertainties. Finally, EEG channel reduction is analyzed toward lightweight XR-SSVEP implementations. As a representative SSVEP-specific DL model, the SSVEP time-frequency fusion network (SSVEP-TFFNet) is evaluated on an open XR benchmark dataset comprising 30 subjects and 1200 trials acquired using Microsoft HoloLens 2. A subject-independent comparison with filter-bank canonical correlation analysis (FBCCA) is performed, while intra- and inter-subject variability are incorporated into the uncertainty evaluation. Results show that SSVEP-TFFNet outperforms FBCCA under the considered XR conditions. Moreover, reduced 6- and 4-channel configurations preserve performance close to the full 8-channel montage. These findings provide evidence of the potential of suitably selected DL models for XR-based SSVEP classification and support uncertainty-aware, reduced-electrode wearable implementations.

## 1. Introduction

Brain–computer interfaces (BCIs) enable direct communication between users and external devices by translating brain activity into control commands [1]. Among non-invasive BCI approaches, electroencephalography (EEG) is the most widely adopted sensing modality, due to its favorable trade-off between cost, portability, temporal resolution, and ease of deployment [2]. These characteristics make EEG particularly suitable for wearable BCI systems [3], where reliable decoding must be achieved while preserving usability outside laboratory environments [4].

Steady-state visually evoked potential (SSVEP) systems represent a widely investigated paradigm for EEG-based BCIs. SSVEPs are periodic EEG responses elicited when a user looks at visual targets, flickering at specific frequency values [5]. In a typical SSVEP-BCI, each visual target is associated with a command and encoded through a distinct modulation frequency, or frequency-related pattern [6]. The user selects the intended command by focusing their attention on the corresponding visual target, while the BCI identifies the attended target by decoding the elicited EEG response. This response is typically more evident over the occipital scalp region and contains discriminative information related to the stimulation pattern, including spectral components at the stimulation frequency and, often, at its harmonics [7]. Thanks to their relatively high signal-to-noise ratio (SNR), limited calibration requirements, and high information transfer rate (ITR), SSVEP-based BCIs are suitable for fast and reliable interaction [8].

Most SSVEP-BCI systems have traditionally relied on computer-screen (CS) stimulation, where visual stimuli are delivered under controlled conditions [9,10]. These setups typically assume fixed viewing distance, stable luminance, controlled background, and well-defined refresh-rate behavior. Such assumptions support repeatable protocols, but limit portability. In this perspective, extended reality (XR) platforms are increasingly relevant, since they allow visual stimuli to be integrated into the user’s surrounding environment through wearable head-mounted displays (HMDs) [11,12,13].

XR-based SSVEP stimulation can support more natural and application-oriented interaction scenarios, with potential benefits in terms of spatial integration, mobility, and user engagement [14,15]. However, it also introduces additional variability with respect to CS setups. The generated stimulation may depend on the display engine, optical-combiner transparency, ambient illumination, contrast reduction, refresh-rate constraints, and interaction between virtual stimuli and the real background. These factors can affect the strength, signal-to-noise ratio, and temporal and spectral characteristics of the elicited SSVEP response, with possible consequences for classification performance [16,17]. Moreover, EEG-HMD co-integration may affect comfort, electrode placement, and signal quality [18,19]. As a consequence, classifier behavior observed under CS stimulation cannot be assumed to transfer directly to XR operating conditions.

Ensuring robust SSVEP classification under these conditions remains an open issue. Conventional methods, such as canonical correlation analysis (CCA) and filter bank canonical correlation analysis (FBCCA) are widely used because of their effectiveness, interpretability, and limited computational burden [20]. More recently, machine-learning (ML) and deep-learning (DL) methods have shown considerable potential, especially with short EEG windows [21]. Nevertheless, such architectures have been designed, trained, and benchmarked primarily on CS-based datasets acquired in controlled settings. Their benchmark validation in XR scenarios remains comparatively limited, despite the different stimulation and measurement conditions introduced by HMD-based platforms [21,22].

Within this context, the first goal of the present work is to investigate the potential of DL-based methods to enhance SSVEP classification in XR scenarios. To this aim, the study focuses on the SSVEP time-frequency fusion network (SSVEP-TFFNet) [22], selected as a representative SSVEP-specific model because it jointly processes time-domain and frequency-domain EEG representations through two parallel branches and a dynamic weighting mechanism. Although originally introduced and validated under conventional CS stimulation, this architecture is particularly relevant for XR-based applications, where platform-dependent effects may influence both the temporal evolution and the spectral content of the elicited EEG response. The present goal is not to generalize the findings to all DL architectures, but to determine whether a suitably selected DL model can provide measurable benefits under XR-based stimulation, thereby offering evidence of the broader potential of DL in this application domain.

Experimental validation is performed on an open XR benchmark dataset comprising 30 subjects and 1200 SSVEP trials acquired using a Microsoft HoloLens 2 device [23]. In this regard, the second goal of the present work is to provide an uncertainty-aware metrological assessment of classifier performance. To this aim, classification accuracy and ITR, widely adopted in SSVEP-BCI studies to quantify target-recognition reliability and command-transmission efficiency, are evaluated within a metrological framework based on the *Guide to the Expression of Uncertainty in Measurement* (GUM) [24]. Rather than being reported as nominal scores, both indicators are treated as measurands derived from a defined experimental and computational procedure and expressed as best estimates with associated standard uncertainties. The uncertainty evaluation accounts for intra-subject variability, related to trial-to-trial fluctuations within each user, and inter-subject variability, related to differences in SSVEP responses across users [25]. This is particularly relevant for XR-based SSVEP systems, where classifier behavior can be affected by platform-dependent stimulation effects, subject-dependent responses, and wearable acquisition conditions. The GUM-based assessment therefore supports a more rigorous comparison between SSVEP-TFFNet and established approaches, as well as a more reliable interpretation of performance robustness under XR-based stimulation.

Finally, the third goal is to conduct an EEG channel-selection analysis to quantify the trade-off between electrode count and classification performance, with the aim of supporting lightweight XR-SSVEP implementations.

The paper is organized as follows. Section 2 provides background on SSVEP classification and states the problem addressed in this work. Section 3 describes the adopted methodology. Section 4 reports the experimental results. Finally, conclusions are drawn and future work is outlined.

## 2. Background and Problem Statement

This section provides the technical background required to define the problems addressed in this work. First, the operating principle of SSVEP-based BCIs is recalled, focusing on the association between visual targets, EEG responses, and command selection. Then, the main SSVEP classification approaches are reviewed, from spectral and correlation-based methods to DL architectures. Finally, the problem statement is formulated around three aspects: DL effectiveness in XR-based SSVEP stimulation, metrology-based performance evaluation, and EEG channel reduction for wearable implementations.

### 2.1. SSVEP-Based Brain–Computer Interfaces

**Figure 1:**
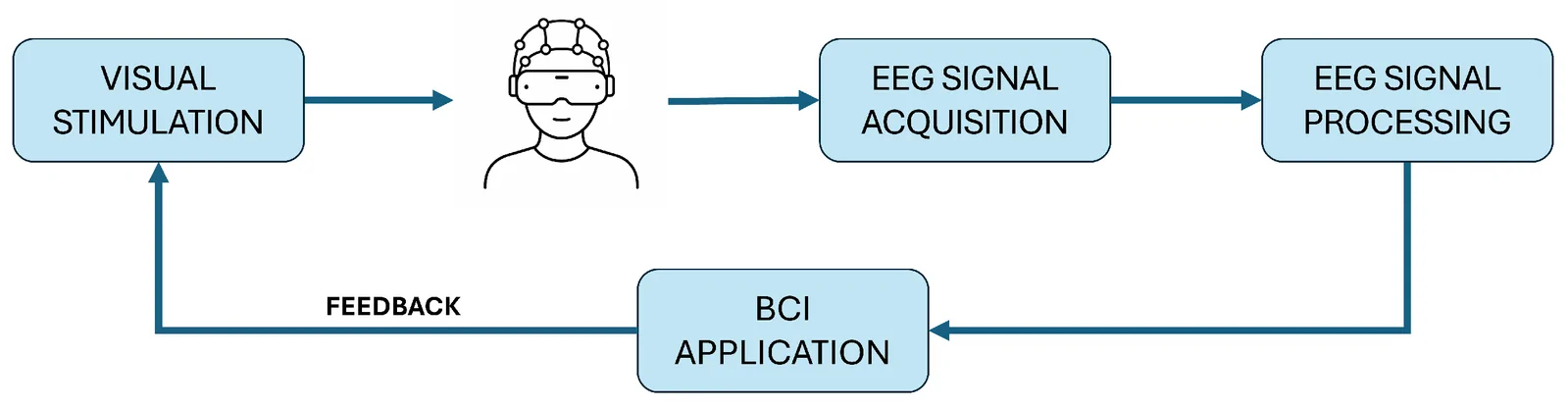
General architecture of an SSVEP-based brain–computer interface.

SSVEP-based BCIs rely on the association between each visual target and a frequency-specific EEG response elicited by periodic stimulation. A typical SSVEP-based BCI architecture is represented in Figure 1.

In a typical implementation, multiple visual targets are presented to the user, each associated with a command and encoded through a distinct modulation frequency, or frequency-related pattern. The user enables the intended command by focusing their attention on the corresponding target, while the BCI decodes the elicited EEG response to infer the selection.

A well-known example of SSVEP-based BCI is the SSVEP speller, where letters or groups of letters are displayed as flickering targets. Each target is associated with a stimulation frequency and corresponds to a spelling command. When the user gazes at the target associated with the desired letter, the elicited response allows the system to identify the selected character and progressively compose words or sentences [26].

From a physiological viewpoint, when a subject attends to a flickering or periodically modulated target, the EEG response exhibits components phase-locked to the stimulation pattern, typically after a latency of about 80–160 ms [27]. SSVEP frequencies are commonly selected in the low- and medium-frequency visual range, approximately from 5 Hz to 40 Hz, to balance response amplitude, comfort, visual fatigue, and display constraints. Lower frequencies generally elicit stronger responses but may increase discomfort, whereas higher frequencies can improve comfort at the cost of reduced amplitude. The response is mainly observed over occipital areas and usually contains a fundamental component at the stimulation frequency, together with harmonics that may provide additional discriminative information. Therefore, SSVEP classification consists of identifying the attended target, and hence the associated command, from the EEG signal acquired during visual stimulation [1].

### 2.2. SSVEP Signal Processing and Classification in XR Environment

SSVEP classification methods exploit the frequency-locked structure of the evoked response. The most intuitive approach is spectral analysis, where the power spectral density (PSD) [12] of the EEG signal is estimated and the attended target is inferred from dominant components associated with the candidate stimulation frequencies. In this case, the classifier searches for spectral peaks at the fundamental frequencies and, when considered, at their harmonics. Although PSD-based methods are simple and interpretable, their performance can be limited by noise, short observation windows, and spectral leakage.

To improve robustness, multichannel methods are commonly adopted. Among them, canonical correlation analysis (CCA) is a widely used calibration-free approach for frequency recognition [13]. CCA compares the EEG signal with sinusoidal references generated at each candidate frequency and its harmonics, and selects the target associated with the highest correlation score. Filter-bank canonical correlation analysis (FBCCA) extends this principle by decomposing the signal into partially overlapping sub-bands and combining the resulting correlation coefficients through predefined weights [23]. This strategy emphasizes informative fundamental and harmonic components and generally improves robustness with respect to standard CCA. For this reason, FBCCA is commonly considered a robust reference method, especially in calibration-free settings. Despite their effectiveness, CCA-based methods rely on fixed sinusoidal templates and linear correlation measures. This assumption is suitable when the elicited response is well represented by stable frequency components, but it may become limiting with short analysis windows, low signal-to-noise ratio (SNR), or variable stimulation and acquisition conditions. In these cases, the EEG response may include subject-dependent, nonlinear, and time-varying characteristics that are not fully captured by predefined references.

To address these limitations, synchronization-based methods have also been investigated. Among them, the multivariate synchronization index (MSI) estimates the degree of synchronization between multichannel EEG signals and frequency-specific reference signals, thereby exploiting phase-related information for SSVEP recognition [11]. A further class of methods includes calibration-based spatial-filtering and template-matching approaches, such as task-related component analysis (TRCA) and its variants [28]. TRCA exploits the reproducibility of task-related EEG components across repeated trials to derive discriminative spatial filters. These methods can achieve high classification performance, although they generally require subject-specific calibration data. Conventional ML approaches have also been applied to SSVEP classification by combining handcrafted temporal, spectral, or spatial features with classifiers such as linear discriminant analysis, support vector machines, or random forests. Their performance, however, strongly depends on the adopted feature-extraction and feature-selection procedures [14,29]. More recently, deep-learning (DL) architectures have attracted increasing attention because they can learn discriminative representations directly from EEG data. Convolutional neural networks (CNNs) [30,31] and long-short-term-memory (LSTM) [32] have shown promising results, especially for short EEG windows where rapid command decoding is required.

The main characteristics of the SSVEP classification strategies discussed in this section are summarized in Table 1. The comparison highlights the trade-off between interpretability, calibration requirements, robustness, and suitability for wearable XR-BCI implementations.

### 2.3. Problem Statement

The methods described above have shown promising performance in their respective application contexts. However, with specific reference to DL, SSVEP classifiers have been evaluated mainly on CS-based datasets, whereas validation on publicly available datasets acquired under XR stimulation remains limited. In light of the display- and acquisition-related differences discussed in the Introduction, performance established under CS-based conditions cannot be assumed to transfer directly to XR environments.

The first problem addressed in this work is therefore to assess on an open XR benchmark dataset whether a suitably selected DL-based SSVEP classifier can outperform a conventional processing strategy. This question is investigated using SSVEP-TFFNet [22], an existing SSVEP-specific architecture previously validated only under CS-based stimulation. Its selection is motivated by its time-frequency design, which combines temporal and spectral EEG representations through parallel processing branches and a fusion mechanism. This structure is particularly relevant for XR conditions, where platform-dependent effects may influence both signal domains. SSVEP-TFFNet is therefore compared with FBCCA, adopted as an established conventional reference method, under the same XR-based acquisition and validation conditions.

The second problem concerns performance interpretation. In SSVEP-BCI studies, accuracy and ITR are commonly used to summarize target-recognition reliability and communication efficiency, respectively. However, nominal values alone may be insufficient when multiple subjects and repeated trials are involved, especially in XR settings where variability may arise from physiological differences and platform-dependent effects. In this work, accuracy and ITR are treated as measurands associated with a defined experimental and computational procedure. They are reported as best estimates with associated standard uncertainties, accounting for intra-subject and inter-subject variability according to a GUM-based framework. This enables more rigorous method comparison and provides a quantitative basis for robustness assessment.

The third problem concerns the number of EEG channels required for effective XR-SSVEP classification. Although multichannel recordings can improve performance by providing richer spatial information, they also increase setup complexity, preparation time, hardware burden, and user discomfort. These aspects are critical for wearable XR-BCIs, where EEG instrumentation must be integrated with an HMD and should remain practical outside laboratory conditions. Therefore, this work evaluates how performance changes when reduced channel subsets are used, with the aim of identifying configurations suitable for lightweight XR-SSVEP implementations.

## 3. Method

**Figure 2:**
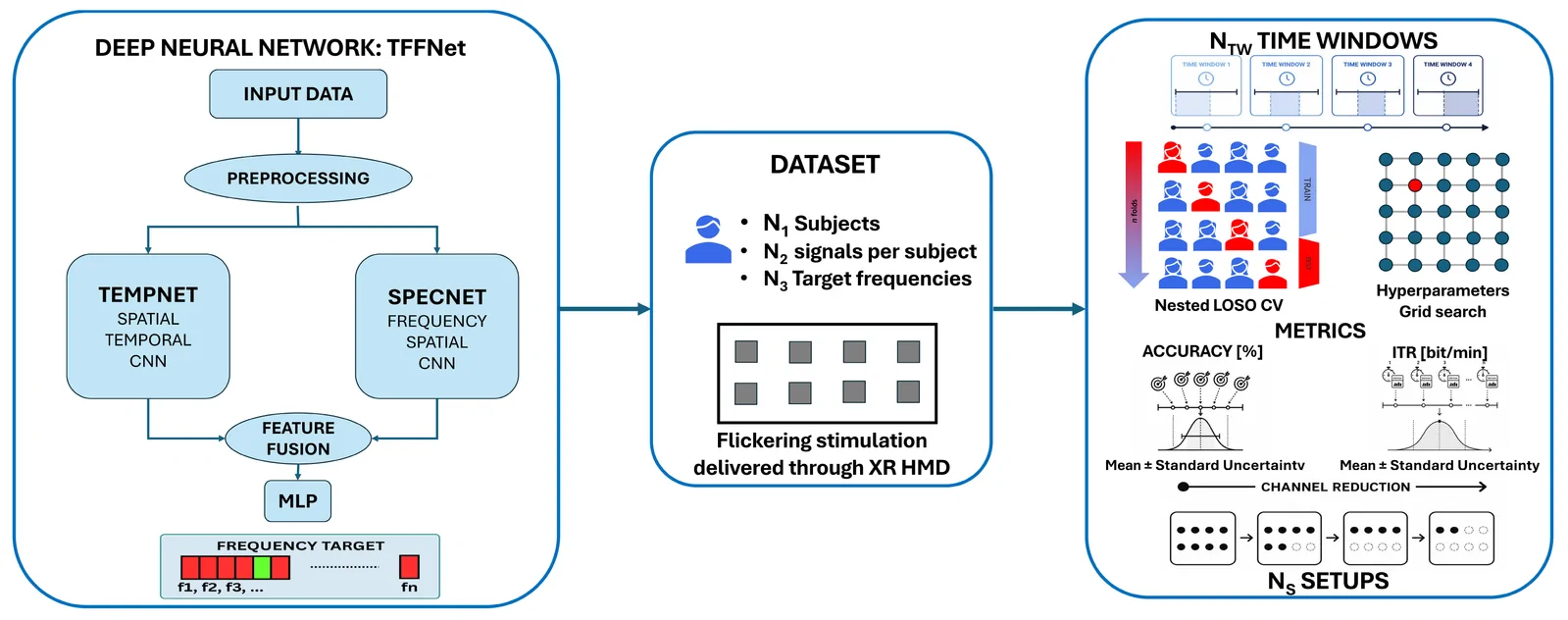
Pipeline of the adopted methodological framework.

This section describes the adopted methodological framework. First, the SSVEP-TFFNet architecture and the corresponding time- and frequency-domain input representations are described. Then, the metrology-based performance evaluation procedure is defined, with classification accuracy and ITR treated as measurands and reported as best estimates with associated standard uncertainties, accounting for intra-subject and inter-subject variability. Finally, a channel-reduction analysis is introduced to assess the feasibility of reduced-electrode XR-SSVEP implementations. For the sake of clarity, the proposed pipeline is shown in Figure 2.

### 3.1. SSVEP-TFFNet

SSVEP-TFFNet is a time-frequency DL architecture introduced for SSVEP classification in [22]. The network jointly processes temporal and spectral EEG representations through two parallel branches, referred to as *TempNet* and *SpecNet*, respectively. The features extracted by the two branches are subsequently processed through a channel-attention mechanism and combined by a feature-fusion module for final target classification.

Each EEG trial is first preprocessed using a fourth-order Butterworth band-pass filter in the 1–45 Hz range. For each analysis window of duration $T_{w}$, the corresponding $N_{T}$ temporal samples are considered, where $N_{T}$ is the integer number of samples associated with $T_{w}$ at the sampling frequency $f_{s}$. Each trial is therefore represented as a multichannel EEG signal $X\in R^{N_{ch}\times N_{T}}$, where $N_{ch}$ is the number of EEG channels included in the considered electrode configuration.

For TempNet, the preprocessed EEG signal is directly used in the time domain and arranged as an input tensor of size $N_{ch}\times N_{T}$. No frequency-domain transformation is applied, so that the temporal ordering of the EEG samples is preserved. TempNet comprises two parallel convolutional paths. In the spatial-temporal path, the first convolutional layer performs spatial filtering using 16 filters, a kernel size of $N_{ch}\times 1$, a stride of 1×1, and zero padding. This is followed by a temporal convolution using 32 filters, a kernel size of $1\times k_{t}$, a stride of 1×2, and zero padding, where $k_{t}$ is selected through the grid-search procedure.

The temporal-spatial path applies the same operations in reverse order. A temporal convolution with kernel size $1\times k_{t}$ is first applied, followed by a spatial convolution with kernel size $N_{ch}\times 1$, extending across all the EEG channels included in the considered configuration. The feature maps obtained from the two paths are then processed by a bidirectional one-dimensional convolutional block. This block includes forward and backward convolutional operations with kernel size 3, where the backward path operates on the temporally reversed feature sequence. Batch normalization and a parametric rectified linear unit (PReLU) activation function are applied after each convolutional layer.

For SpecNet, the same preprocessed EEG signal is transformed by means of the fast Fourier transform (FFT), computed independently for each channel. Differently from magnitude-only spectral representations, the adopted representation preserves both the real and imaginary parts of the complex spectrum. This allows the network to exploit amplitude- and phase-related information, which can be relevant for SSVEP decoding, especially when frequency- and phase-dependent stimulation patterns are involved [22,33].

The frequency-domain representation is restricted to the 7–45 Hz range, which includes the fundamental stimulation frequencies of the considered dataset and their relevant harmonic components. Let $N_{F}$ denote the number of frequency bins retained within this interval. The real and imaginary parts of the corresponding FFT coefficients are concatenated along the spectral dimension, resulting in a real-valued input tensor $X_{F}\in R^{N_{ch}\times 2N_{F}}$. Thus, the spectral representation preserves the same number of EEG channels as the temporal input, while its second dimension depends on the number of retained frequency coefficients.

SpecNet also comprises two parallel convolutional paths. In the spatial-spectral path, spatial filtering is first performed using 32 filters and a kernel size of $N_{ch}\times 1$, followed by a spectral convolution with kernel size $1\times k_{s}$, where $k_{s}$ is selected through the grid-search procedure. In the spectral-spatial path, the same operations are applied in reverse order. Both paths also include a long spectral convolution with kernel size 1×35 to capture broader frequency-domain patterns. As in TempNet, batch normalization and PReLU activation are applied after each convolutional layer.

The temporal and spectral representations extracted by TempNet and SpecNet are subsequently processed through the channel-attention mechanism and combined in the feature-fusion stage. The fusion module adaptively weights the contributions of the two branches and provides the feature vector used by the final classification layer. The network output is the predicted SSVEP class, corresponding to one of the stimulation targets $\left(f_{1},f_{2},\ldots ,f_{M}\right)$. Since the spatial convolutional kernels extend over $N_{ch}$ channels, the architecture can be adapted to the different electrode configurations considered in the channel-reduction analysis.

### 3.2. Metrology-Based Performance Evaluation

As aforementioned, the performance evaluation of SSVEP-TFFNet is carried out according to the GUM framework [24]. The first step consists in defining the measurands of interest. In this work, the considered measurands are classification accuracy and ITR, which are widely used in SSVEP-BCI studies to quantify target-recognition reliability and command-transmission efficiency, respectively.

Classification accuracy Acc is defined as the ratio between the number of correctly classified targets $N_{c}$ and the total number of targets observed by the user $N_{t}$ within a specific set of trials. Defined in this way, classification accuracy is a single value and does not account for the variability observed across repeated sets of trials performed by the same user. Therefore, for each subject, repeated trials are considered as repeated observations of the measurand. Let $Acc_{s,n}$ denote the accuracy value obtained from the *n*-th set of trial, or trial group, of the *s*-th subject, with $n=1,\ldots ,N_{s}$, where $N_{s}$ is the number of repeated sets of trials available for that subject. The best estimate of the classification accuracy for subject *s* is then obtained as the arithmetic mean ${\bar{Acc}}_{s}$ among the repeated sets of trials. The standard uncertainty associated with ${\bar{Acc}}_{s}$ is evaluated through a Type A uncertainty evaluation, since it is estimated from repeated observations. It represents the degree of uncertainty with which the arithmetic mean estimates the subject-specific measurand:

(1) $$u_{\mathrm{intra},s}\left(Acc\right)=\sqrt{\frac{1}{N_{s}\left(N_{s}-1\right)}\sum_{n=1}^{N_{s}}{\left(Acc_{s,n}-{\bar{Acc}}_{s}\right)}^{2}}$$

This contribution accounts for intra-subject variability, namely the variability associated with repeated sets of trials performed by the same user. However, EEG-based BCI performance is also affected by relevant inter-subject variability, due to differences in physiological responses, attention, visual perception, and signal quality across users. Therefore, the measurement of classification accuracy is repeated over a set of *S* subjects. Each subject provides a subject-specific best estimate ${\bar{Acc}}_{s}$ and a corresponding intra-subject standard uncertainty $u_{\mathrm{intra},s}\left(Acc\right)$. The overall best estimate of classification accuracy $\bar{Acc}$ is computed as the arithmetic mean of the subject-specific estimates: The inter-subject uncertainty contribution accounts for the variability of the subject-specific mean accuracies around the overall best estimate. It is evaluated as the standard uncertainty of the mean of the subject-specific estimates:

(2) $$u_{\mathrm{inter}}\left(Acc\right)=\sqrt{\frac{1}{S(S-1)}\sum_{s=1}^{S}{\left({\bar{Acc}}_{s}-\bar{Acc}\right)}^{2}}$$

The intra-subject contribution is obtained by combining the subject-specific intra-subject uncertainty contributions. Following a variance-based decomposition, it is computed as the square root of the mean of the subject-specific variances:

(3) $$u_{\mathrm{intra}}\left(Acc\right)=\sqrt{\frac{1}{S}\sum_{s=1}^{S}u_{\mathrm{intra},s}^{2}\left(Acc\right)}$$

Assuming independent intra-subject and inter-subject contributions, the combined standard uncertainty associated with classification accuracy is obtained as

(4) $$u\left(Acc\right)=\sqrt{u_{\mathrm{inter}}^{2}\left(Acc\right)+u_{\mathrm{intra}}^{2}\left(Acc\right)}$$

Accordingly, classification accuracy is reported as $Acc=\bar{Acc}\pm u\left(Acc\right)$.

The second measurand considered in this work is the ITR, which quantifies the amount of information conveyed per unit time. It is computed as

(5) $$ITR=\left[\log_{2}\left(M\right)+Acc\log_{2}\left(Acc\right)+\left(1-Acc\right)\log_{2}\left(\frac{1-Acc}{M-1}\right)\right]\frac{60}{T_{w}}$$

where *M* is the number of possible targets, or classes, and $T_{w}$ is the time window containing the EEG samples. In the proposed procedure, *M* is fixed by the experimental paradigm and is therefore treated as a quantity without associated uncertainty. Similarly, $T_{w}$ is treated as fixed for each considered analysis condition, since the evaluation is repeated for different time windows. Consequently, the uncertainty associated with ITR is obtained by propagating the uncertainty of classification accuracy through the ITR model according to the law of propagation of uncertainty. The best estimate of ITR ($\bar{ITR}$) is obtained by evaluating (5) at the best estimate of classification accuracy, while its standard uncertainty is evaluated through the Law of Propagation of Uncertainty [24] as

(6) $$u\left(ITR\right)={\left|\frac{\partial ITR}{\partial Acc}\right|}_{Acc=\bar{Acc}}u\left(Acc\right)$$

Therefore, ITR is reported as $ITR=\bar{ITR}\pm u\left(ITR\right)$. This formulation allows ITR to inherit the uncertainty contributions associated with classification accuracy. Therefore, the reported ITR uncertainty accounts for both intra-subject variability (related to repeated sets of trials within each user) and inter-subject variability (related to differences across users).

Overall, this *metrology-based* choice of reporting the standard uncertainty rather than the more commonly used sample standard deviation reflects the different information conveyed by the two parameters. While the sample standard deviation characterizes the dispersion of the individual performance values across repeated trials or subjects, the standard uncertainty characterizes the uncertainty associated with the corresponding mean value adopted as the best estimate of the measurand. Therefore, the proposed formulation does not replace conventional descriptive statistics or statistical testing, but complements them by quantifying how precisely the mean classification performance is estimated. From a model-evaluation perspective, this allows nominal differences between classifiers or experimental configurations to be interpreted together with the uncertainty associated with their estimates. From a decision-making perspective, it provides additional quantitative support when selecting a classifier, an analysis window, or a reduced-channel configuration, since small performance differences should be interpreted more cautiously when they are comparable with the associated uncertainty. Conversely, improvements that remain appreciable relative to the reported uncertainty provide stronger evidence in support of a specific design choice.

### 3.3. Channel-Reduction Analysis

For wearable XR-BCI systems, EEG configurations should not only provide adequate classification performance, but should also remain practical to deploy. In this regard, the number of EEG channels is a critical design parameter. A larger montage can provide richer spatial information and improve SSVEP classification, but it also increases hardware complexity, setup time, sensor encumbrance, and user discomfort. These aspects become particularly relevant when the EEG system must be co-integrated with an HMD and used outside laboratory conditions. To investigate this trade-off, a channel-reduction analysis is carried out by progressively reducing the EEG montage from the full electrode configuration. For each configuration, the same preprocessing, input construction, validation strategy, and metrology-based performance evaluation are applied, so that the effect of channel reduction is assessed under comparable experimental conditions. For each configuration, accuracy and ITR are reported as best estimates with associated standard uncertainties, following the GUM-based procedure described above.

From a practical perspective, reducing the number of EEG channels can shorten preparation time, decrease the amount of conductive gel or saline required, and simplify electrode placement, cabling, and acquisition hardware. It can also reduce system weight, power consumption, and mechanical interference between the EEG sensors and the HMD, thereby improving user comfort and facilitating repeated or prolonged use outside controlled laboratory environments. Moreover, a reduced electrode configuration decreases the amount of data to be acquired, transmitted, and processed, which is beneficial for real-time implementations executed directly on the XR device, on a companion unit, or on an edge-computing platform. The resulting comparison therefore quantifies not only the trade-off between electrode count and classification performance, but also the extent to which system wearability and deployment practicality can be improved. In particular, the preservation of performance with the 6- and 4-channel configurations would indicate that a substantial reduction in setup and hardware burden can be achieved without a substantial loss in classification performance, supporting the development of lightweight XR-SSVEP implementations.

## 4. Experimental Results

This section reports the experimental validation of SSVEP-TFFNet on the considered XR-based SSVEP benchmark. First, the dataset and the adopted validation strategy are described. Then, the classification performance of SSVEP-TFFNet is compared with FBCCA in terms of classification accuracy and ITR for different EEG time windows. Finally, the channel-reduction analysis is reported to assess the effect of using reduced EEG montages.

### 4.1. Dataset Description

The performance assessment was carried out on the open XR-based SSVEP benchmark dataset developed in [23]. The dataset includes EEG recordings from S=30 healthy subjects, composed of 16 males and 14 females, aged between 21 and 34 years. Visual stimulation was delivered through a Microsoft HoloLens 2 HMD, which allowed the visual targets to be presented in an XR environment during a visually cued SSVEP target-selection task. The user interface consisted of eight square visual targets arranged in a 2×4 matrix and positioned at a virtual distance of 1.0 m from the user. Each visual target flickered at a distinct frequency between 8 Hz and 15 Hz, with 1 Hz spacing. Therefore, the task was formulated as an M=8 class classification problem, with each class corresponding to one stimulation frequency and to the associated visual target.

Each trial consisted of a 5 s stimulation interval, during which all visual targets flickered simultaneously while the subject focused attention on the cued target. The experimental protocol included five acquisition cycles for each subject. In each cycle, the eight visual targets were sequentially cued once. Therefore, each subject performed 40 trials, corresponding to 8 visual targets repeated over 5 cycles. Overall, the dataset comprised 1200 trials. EEG signals were acquired using a g.tec Unicorn Hybrid Black wearable headset, operating at a sampling frequency of 250 Hz with 24-bit resolution. The recordings were obtained from eight electrodes placed over the occipital region according to the international 10–20 system: PO7, PO3, POz, PO4, PO8, O2, Oz, and O1. Reference and ground electrodes were located on the mastoids. In this work, the acquired EEG trials were processed according to the pipeline described in Section 3. Although the present validation is based on a single dataset and XR device, to the best of the authors’ knowledge, the adopted dataset was introduced as the first publicly available benchmark specifically designed for SSVEP signals elicited through XR-based stimulation [23]. Its public availability enables reproducible comparisons among classification methods under common stimulation and acquisition conditions. Moreover, the proposed processing, validation, and metrology-based assessment procedures are not intrinsically tied to the Microsoft HoloLens 2 and can, in principle, be applied to other public XR-based SSVEP datasets. Nevertheless, the numerical performance reported in this study remains specific to the considered device and experimental protocol. Therefore, further cross-device and cross-dataset validation is required to assess broader generalizability.

### 4.2. Validation Strategy

To obtain performance estimates suitable for the intra-subject and inter-subject uncertainty evaluation described in Section 3, a nested leave-one-subject-out (LOSO) cross-validation strategy was adopted for both SSVEP-TFFNet and the reference FBCCA method. The outer LOSO loop was used to estimate generalization performance on unseen subjects. At each outer fold, all trials belonging to one subject were held out as the test set, while the remaining S−1 subjects were used for training and model selection. Since the dataset includes S=30 subjects, the outer loop consisted of 30 folds. This procedure ensured that no data from the test subject were used during either training or hyperparameter selection. Within each outer fold, an inner validation loop was applied to the non-test subjects. This inner loop was used for model selection, including the optimization of the SSVEP-TFFNet hyperparameters and the configuration of the reference classifier under the same subject-independent evaluation principle. For SSVEP-TFFNet, a grid-search procedure was performed over the hyperparameter space reported in Table 2. The hyperparameter configuration yielding the highest validation accuracy in the inner loop was selected and then used to train the model evaluated on the held-out subject of the corresponding outer fold. Hence, for each outer fold, classification accuracy was evaluated from the repeated trials of the held-out subject. The subject-specific estimates and the corresponding uncertainty contributions were then combined according to the metrology-based procedure described in Section 3. The same procedure was applied for each considered EEG time window.

With specific reference to SSVEP-TFFNet, the network was trained for 150 epochs using the Adam optimizer and the categorical cross-entropy loss function with a label-smoothing factor of 0.1. No early-stopping criterion was adopted, and all models were trained for the predefined number of epochs. Training, hyperparameter optimization, nested validation, and uncertainty assessment were performed entirely offline and are not part of the online processing chain. During deployment, only EEG preprocessing and forward inference through the trained network are required. Depending on the computational resources available, these operations may be executed directly on the XR device, on a companion computing unit, or on an edge-computing platform connected to the wearable system. Therefore, the adopted validation and metrology-based assessment procedures do not introduce an additional computational burden during online use.

### 4.3. Classification Performance

The classification performance of SSVEP-TFFNet was evaluated for EEG time windows $T_{w}$ of 0.50 s, 0.75 s, 1.00 s, and 1.25 s. Results were compared with FBCCA, used as the baseline SSVEP classification method. For each time window, classification accuracy and ITR were reported as best estimates with associated standard uncertainties. As described, for each subject, the 40 available trials were organized into five sets of trials, denoted as acquisition cycles, each including one presentation of the eight stimulation targets. Each cycle therefore provided one accuracy value, computed over eight target presentations. Consequently, five accuracy values were obtained for each subject. Their arithmetic mean was adopted as the subject-specific best estimate, while the corresponding intra-subject standard uncertainty was evaluated according to Equation (1).

The same procedure was repeated for all 30 subjects. The overall best estimate of accuracy was then obtained as the mean of the subject-specific estimates. The inter-subject standard uncertainty was evaluated from the variability of the 30 subject-specific mean accuracies according to Equation (2), while the overall intra-subject contribution was obtained by combining the subject-specific intra-subject uncertainty contributions. The two components were finally combined according to Equation (4) to obtain the standard uncertainty associated with the overall accuracy estimate. For each time window, the corresponding ITR best estimate was obtained by evaluating the conventional ITR model at the overall best estimate of classification accuracy. Its standard uncertainty was then derived by propagating the combined uncertainty of accuracy through the ITR model according to Equation (6).

**Figure 3:**
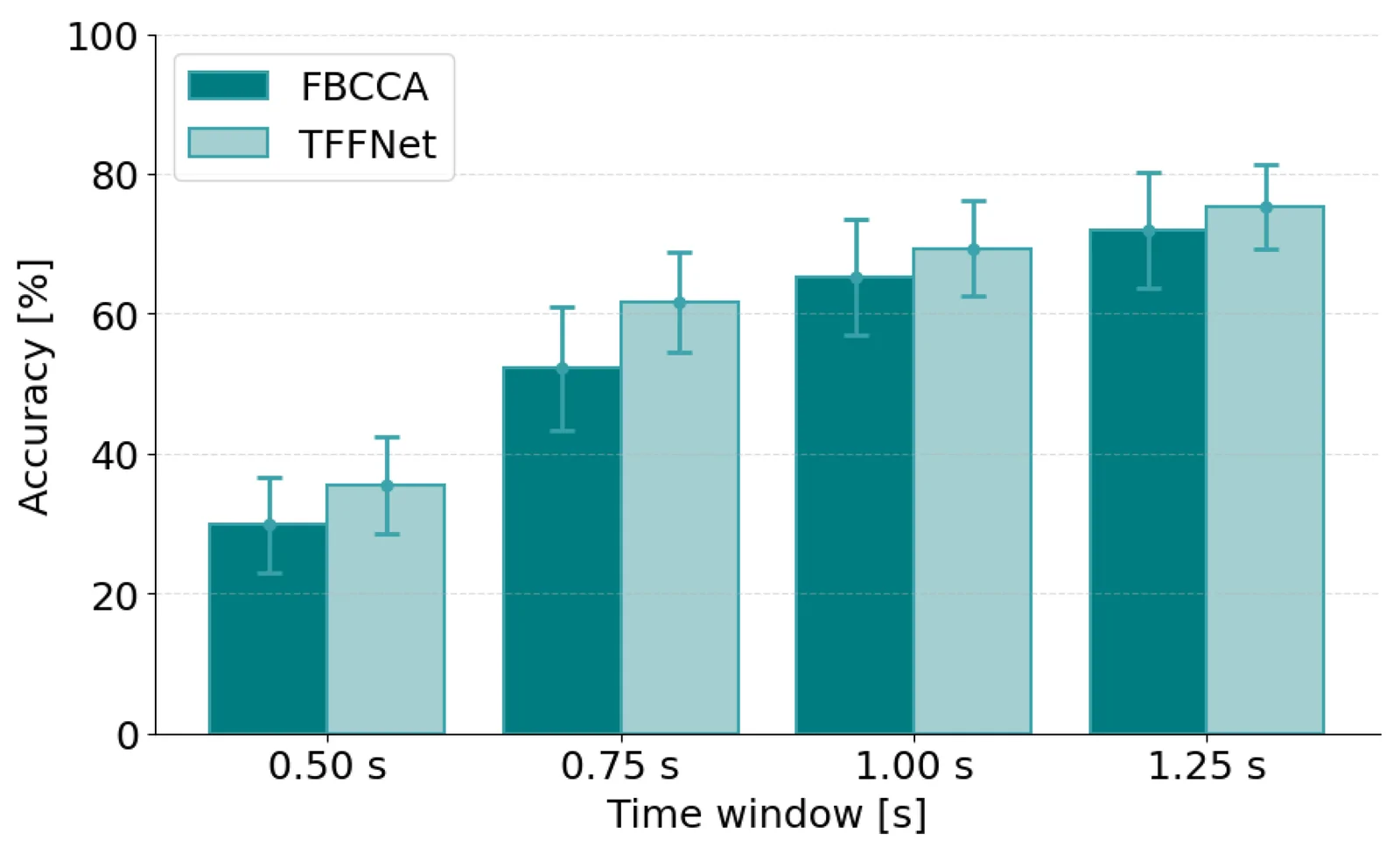
Classification accuracy achieved by SSVEP-TFFNet and FBCCA for EEG analysis windows of 0.50 s, 0.75 s, 1.00 s, and 1.25 s. Results are reported as best estimates with associated standard uncertainty values.

**Figure 4:**
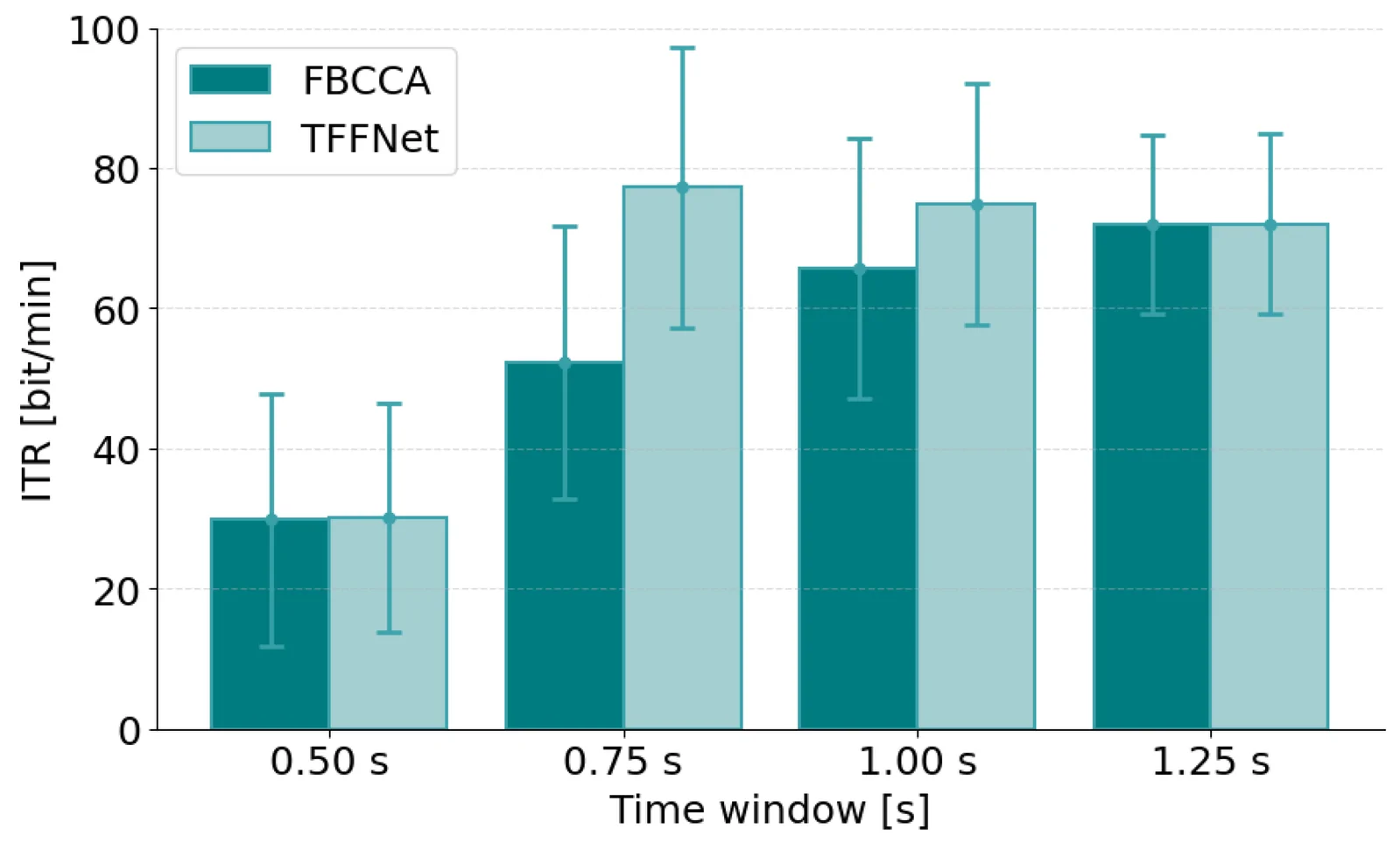
ITR achieved by SSVEP-TFFNet and FBCCA for EEG analysis windows of 0.50 s, 0.75 s, 1.00 s, and 1.25 s. Results are reported as best estimates with associated standard uncertainty values.

Figure 3 and Figure 4 report the classification accuracy and ITR obtained by SSVEP-TFFNet and FBCCA across the considered time windows. SSVEP-TFFNet outperformed FBCCA for all values of $T_{w}$. The maximum ITR achieved by SSVEP-TFFNet was observed at $T_{w}=0.75$ s, with a value of (77.2±20.1) bit/min, compared with (54.8±19.5) bit/min for FBCCA. This corresponds to an ITR increase of 22.6 bit/min. These results suggest that the time-frequency representation learned by SSVEP-TFFNet is particularly effective for intermediate analysis windows, where the EEG signal is long enough to contain discriminative SSVEP information while still preserving high command-transmission speed.

**Figure 5:**
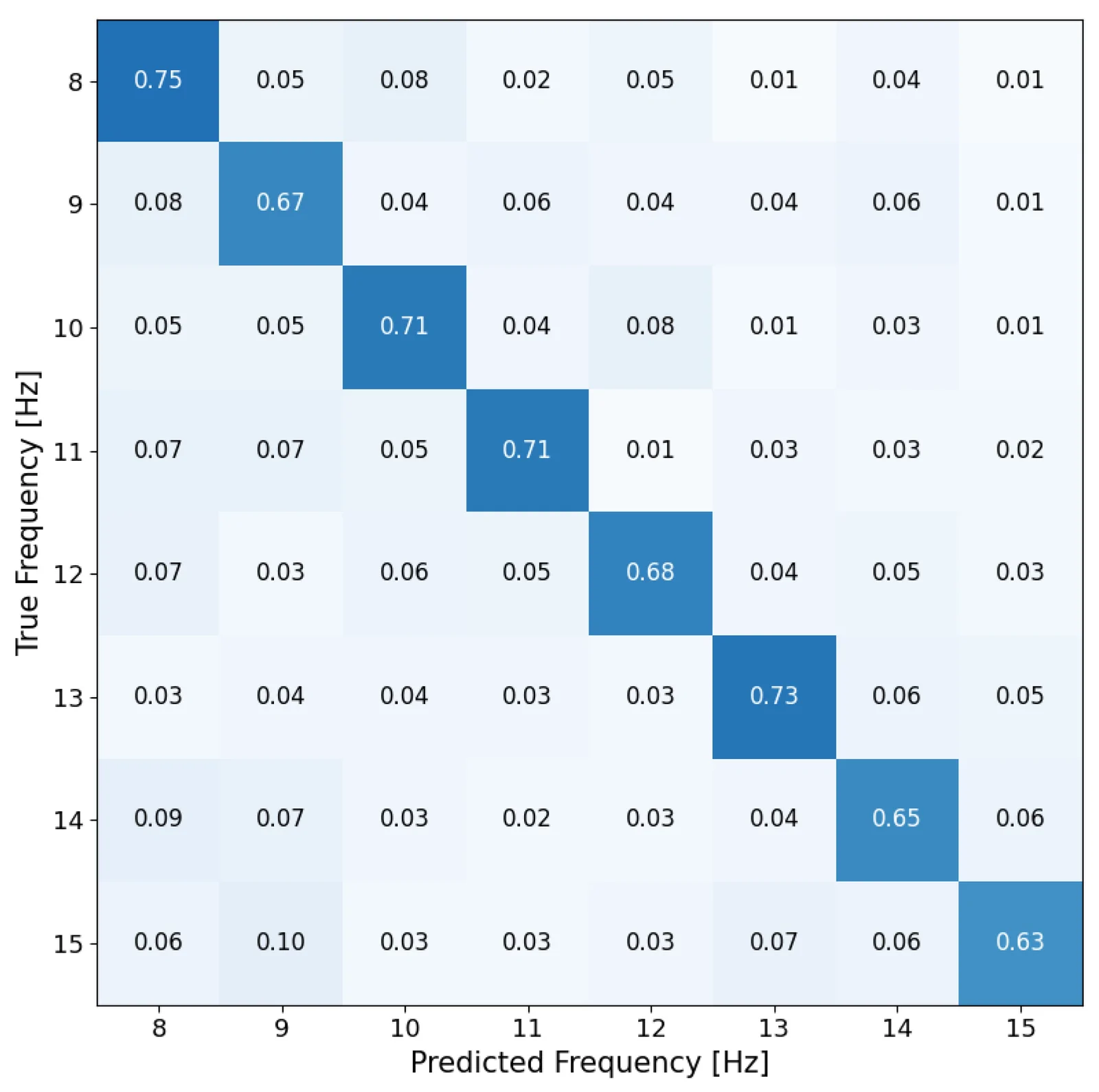
Overall confusion matrix obtained for $T_{w}=0.75$ s, aggregated across all subjects.

**Figure 6:**
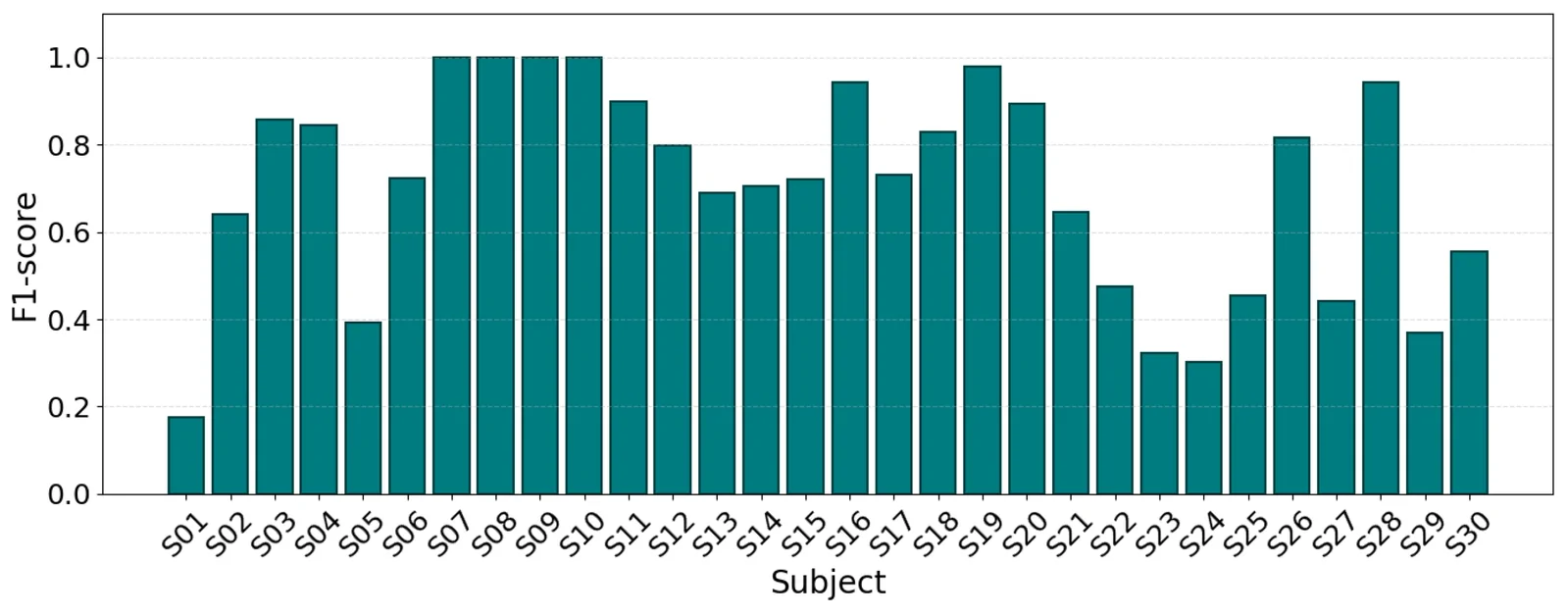
Subject-wise F1-scores obtained for $T_{w}=0.75$ s.

**Figure 7:**
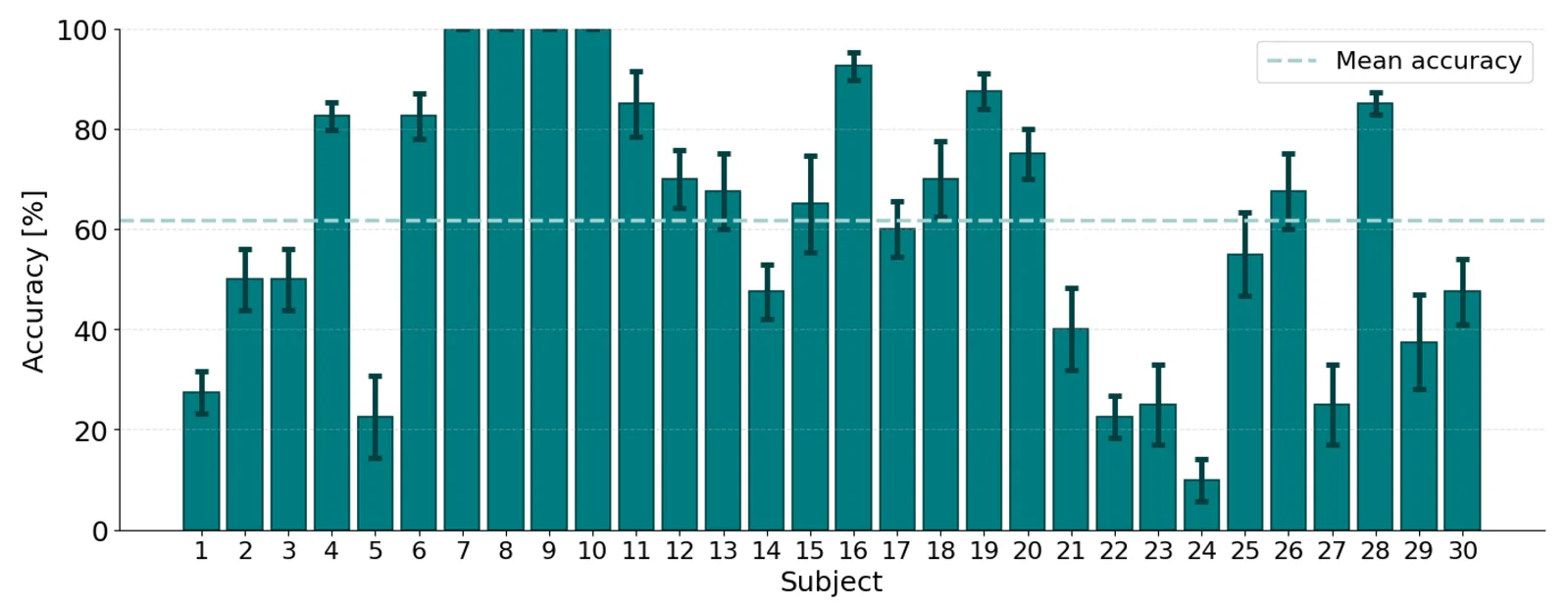
Subject-wise classification accuracy obtained for $T_{w}=0.75$ s. Each bar represents the mean accuracy ± standard uncertainty, while the dashed horizontal line indicates the overall best estimate across all 30 subjects.

Additionally, for $T_{w}=0.75$ s, Figure 5 and Figure 6 report the overall confusion matrix, aggregated across all subjects, and the subject-wise F1-scores, respectively. These results complement the accuracy and ITR analysis by highlighting class-specific misclassification patterns and inter-subject variability in classification performance. Figure 7 further provides a subject-wise overview of classification accuracy, including the corresponding intra-subject uncertainty contributions and the overall inter-subject performance estimate for the same time window.

To assess whether the performance differences between SSVEP-TFFNet and FBCCA were statistically significant, a subject-wise paired comparison was performed separately for each EEG time window. For each subject and time window, the statistical analysis considered the subject-specific mean accuracy obtained from the five acquisition cycles. The associated standard uncertainties were not included as additional observations in the hypothesis tests, since they quantify the uncertainty associated with each subject-specific estimate rather than independent realizations of classifier performance. The normality of the paired differences between the subject-specific mean accuracies was assessed using the Shapiro–Wilk test. Since the normality assumption was rejected (p<0.05, α=0.05), the non-parametric Wilcoxon signed-rank test was adopted. No correction for multiple comparisons was applied, since the tests were defined *a priori* and each time window represents a distinct operating condition associated with a specific hypothesis. Accordingly, the resulting *p*-values were interpreted separately for each time window and were not used to support a single global statistical claim across all conditions. A statistically significant difference was observed for $T_{w}=0.75$ s (p=0.005, α=0.05). This result indicates that the adopted DL classifier significantly outperformed FBCCA for the $T_{w}=0.75$ s window in the considered XR-based setup.

### 4.4. Channel-Reduction Assessment

**Figure 8:**
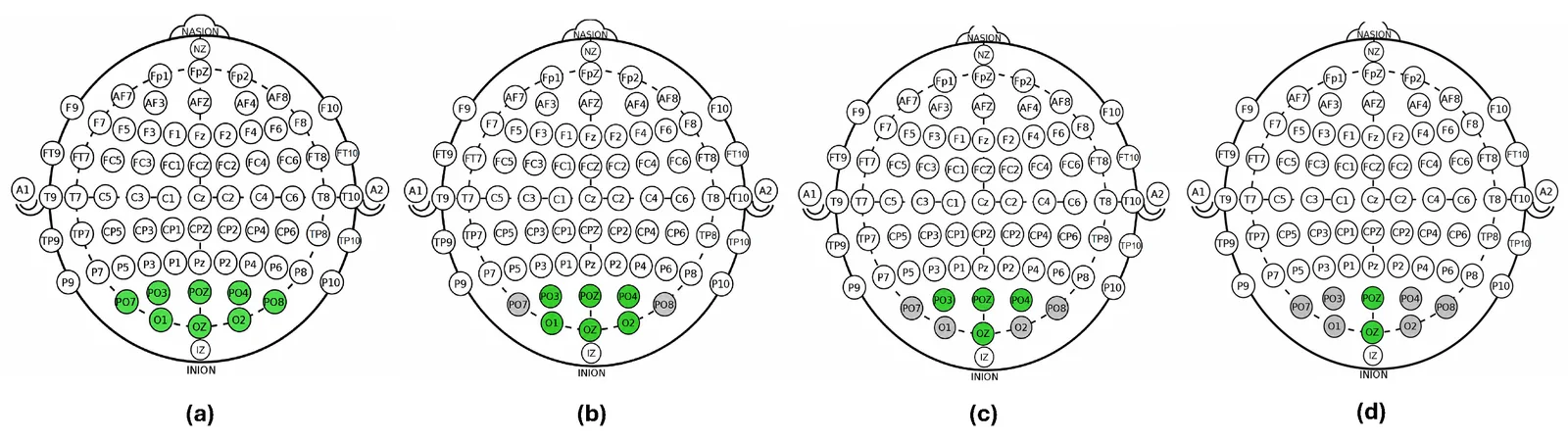
A 10–20-system representation of the acquisition setups considered for the channel-reduction task. Starting from the initial (**a**) 8-channel setup, the derived configurations are the (**b**) 6-channel, (**c**) 4-channel, and (**d**) 2-channel setups.

Starting from the full 8-channel occipital montage, progressively reduced configurations were evaluated to quantify the effect of decreasing the number of electrodes on classification performance. The considered channel configurations are reported in Table 3 and illustrated in Figure 8. Although the selected subsets may appear heuristic, they were defined according to established physiological knowledge of SSVEP generation, which is predominantly associated with occipital and parieto-occipital regions. Accordingly, the reduction procedure preserved the central electrodes Oz and POz, where SSVEP activity is typically more pronounced, while progressively removing the more peripheral channels. The aim was not to identify an optimal channel subset through an automated selection procedure, but to evaluate the performance-wearability trade-off using simple, physiologically meaningful configurations that can be readily implemented in wearable XR-BCI systems.

In this analysis, the full grid-search procedure was not repeated for each channel subset. Instead, for each time window, the most recurrent hyperparameter configuration selected during the nested LOSO validation on the full-channel setup was adopted. This choice was made to isolate the effect of electrode reduction from hyperparameter re-optimization and to keep the comparison among channel configurations under controlled conditions. For $T_{w}=0.75$ s and $T_{w}=1.25$ s, the adopted configuration consisted of a learning rate of 0.01, batch size of 128, dropout rate of 0.5, TempNet kernel size of 10, and SpecNet kernel size of 8. For $T_{w}=1.00$ s, the same configuration was used, except for the SpecNet kernel size, which was set to 10.

**Figure 9:**
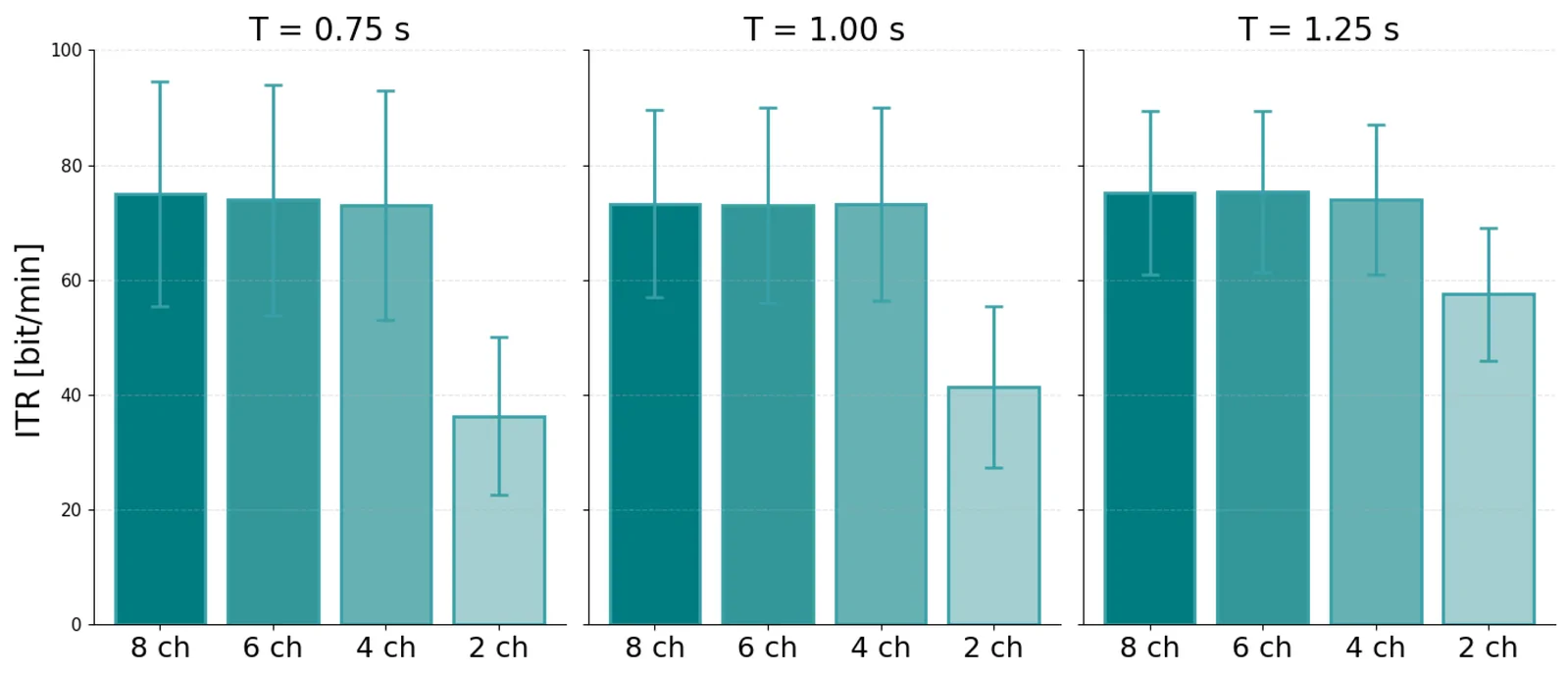
ITR achieved by SSVEP-TFFNet for the considered channel configurations. Results are reported in bit/min as best estimates with associated standard uncertainty values.

For each electrode configuration, the same classification and metrology-based evaluation procedure was applied. Results are shown in Figure 9 in terms of ITR, reported as best estimate with associated standard uncertainty. As shown, the 6-channel and 4-channel configurations provided performance close to that of the full montage. In particular, for the 0.75 s time window, the 6-channel configuration, including PO3, POz, PO4, O2, Oz, and O1, achieved an ITR of (73.9±20.0) bit/min, while the 4-channel configuration, including PO3, POz, PO4, and Oz, achieved (73.0±19.9) bit/min. Both values were within 2 bit/min of that obtained with the full 8-channel configuration, which reached (75.0±19.6) bit/min. The preservation of performance with 6 and 4 channels can be reasonably attributed to the fact that these configurations retain electrodes over the central occipital and parieto-occipital regions, where SSVEP activity is typically strongest, while also preserving a certain degree of lateral spatial coverage and inter-channel redundancy. Therefore, the spatial filters implemented by SSVEP-TFFNet can still exploit complementary information across multiple recording locations, even after removing the more peripheral electrodes.

Conversely, the 2-channel configuration, composed only of POz and Oz, showed a more marked performance reduction, with an ITR of (36.3±13.8) bit/min. Although these electrodes are located over highly relevant SSVEP-responsive areas, restricting the montage to two midline channels substantially reduces lateral spatial information, channel diversity, and redundancy against local noise or subject-dependent variations. It also limits the amount of spatial structure available to the convolutional layers, whose kernels extend across the considered EEG channels. As a result, the network has less information available to discriminate among the stimulation classes.

These findings indicate that a moderate reduction in the number of electrodes can preserve performance close to that of the full montage, whereas an excessively reduced configuration may remove relevant spatial information. This interpretation is consistent with the role of occipital and parieto-occipital regions in SSVEP generation, although a dedicated channel-contribution or automated channel-selection analysis would be required to determine the optimal subset definitively.

The effect of channel reduction was also assessed using the Wilcoxon signed-rank test (α=0.05). Analogously to the comparison between SSVEP-TFFNet and FBCCA, the statistical analysis was performed using the subject-specific mean accuracies obtained from the five acquisition cycles, whereas the associated standard uncertainties were not treated as additional observations. For each time window, the full 8-channel configuration was separately compared with the predefined 6-, 4-, and 2-channel configurations. No correction for multiple comparisons was applied, since the comparisons were defined *a priori* and addressed distinct hypotheses concerning each reduced configuration with respect to the full-channel reference. Moreover, no exhaustive post-hoc comparison among all possible channel configurations was performed, and the resulting *p*-values were interpreted separately for each predefined comparison. No statistically significant differences were observed between the 8-channel configuration and the 6- or 4-channel configurations for any of the considered time windows (p>0.05). Conversely, the 2-channel configuration showed a statistically significant performance reduction at all time windows (p<0.05). These results support the conclusion that a moderate reduction in the number of EEG channels can improve wearability and simplify the acquisition setup without introducing a statistically significant performance penalty. In contrast, the 2-channel configuration appears more critical, as it leads to a significant reduction in classification accuracy and, consequently, in ITR.

## 5. Conclusions

In this work, the applicability of DL-based SSVEP classification in wearable XR-BCI scenarios was investigated. In particular, SSVEP-TFFNet was evaluated on an open XR benchmark dataset acquired using a Microsoft HoloLens 2 device and compared with FBCCA as a traditional baseline method.

A GUM-based metrological framework was adopted to evaluate classification accuracy and ITR as measurands. Both quantities were reported as best estimates with associated standard uncertainties, accounting for intra-subject and inter-subject variability. This *metrology-based* formulation allowed classifier performance to be interpreted beyond nominal values, which is particularly relevant in XR settings where subject-dependent responses and platform-dependent stimulation conditions may affect decoding reliability.

Results showed that SSVEP-TFFNet achieved higher performance estimates than FBCCA across the considered time windows, with a statistically significant improvement at $T_{w}=0.75$ s. Although the findings are specific to the considered architecture, dataset, and XR device, they provide evidence that suitably selected DL models can improve SSVEP classification in XR-based environments. The highest ITR was obtained for the short time window $T_{w}=0.75$ s, supporting the relevance of short-window decoding for fast command transmission in wearable SSVEP-BCIs.

Finally, the channel-reduction analysis showed that the 6- and 4-channel configurations preserved performance close to the full-channel configuration without statistically significant reductions. Conversely, the 2-channel configuration produced a marked performance decrease, indicating that excessive channel reduction may remove relevant spatial information. These results support the feasibility of reduced-electrode XR-SSVEP implementations capable of improving wearability and simplifying the acquisition setup.

Future work will extend the validation to additional DL architectures, publicly available XR datasets, XR devices, and real-time closed-loop BCI experiments. A further direction will be the investigation of automated channel-selection strategies and the propagation of uncertainty along the whole metrological chain, from EEG acquisition and input signal representation to DL model processing and final classification outcome.

## Figures and Tables

**Table 1:** Qualitative comparison of the main SSVEP classification strategies.

Method	Main Principle	Calibration	Strengths	Limitations
PSD-based methods	Detection of spectral peaks at the stimulation frequencies and their harmonics	Calibration-free	Simple, interpretable, and characterized by low computational complexity	Sensitive to noise, spectral leakage, short analysis windows, and inter-subject variability
CCA	Correlation between multichannel EEG signals and sinusoidal reference signals	Calibration-free	Robust multichannel frequency recognition; widely adopted baseline	Relies on fixed sinusoidal templates and linear correlation measures
FBCCA	CCA applied to multiple filter-bank sub-bands, followed by weighted score fusion	Calibration-free	Improved exploitation of fundamental and harmonic components; strong reference method	Requires predefined sub-band decomposition and weighting parameters
MSI-based methods	Estimation of multivariate synchronization between EEG signals and frequency-specific reference signals	Calibration-free	Exploits phase and synchronization information; suitable for multichannel SSVEP recognition	Performance may depend on signal quality, window duration, and reference-signal design
TRCA-based methods	Spatial filtering based on the reproducibility of task-related EEG components across trials	Calibration-based	High performance when subject-specific calibration data are available	Requires repeated calibration trials and subject-specific model estimation
ML-based methods	Classification of handcrafted temporal, spectral, or spatial features using models such as LDA, SVM, or random forests	Data-dependent	Flexible classification framework; lower computational burden than many DL architectures	Performance depends on feature engineering, feature selection, and representative training data
DL-based methods	End-to-end learning of discriminative temporal, spectral, and spatial EEG representations	Data-dependent	Can model nonlinear and complex signal patterns; suitable for short-window decoding	Requires sufficient data, careful validation, and comparatively higher computational resources

**Table 2:** Hyperparameter space considered for the grid-search procedure within the inner loop of the nested LOSO cross-validation.

Hyperparameter	Values
Learning rate	0.01, 0.001, 0.0001
Batch size	32, 128
Dropout rate	0.3, 0.5
Kernel size TempNet	10, 15
Kernel size SpecNet	8, 10

**Table 3:** EEG channel selection for the channel-reduction assessment.

Number of Channels	Channels
8	PO7, PO3, POz, PO4 PO8, O2, Oz, O1
6	PO3, POz, PO4 O2, Oz, O1
4	PO3, POz PO4, Oz
2	POz, Oz

## Data Availability

Data can be made available upon request.
